# Task success in trained spiking neural network models coincides with emergence of cross-stimulus-modulated inhibition

**DOI:** 10.1007/s00422-025-01030-4

**Published:** 2026-01-07

**Authors:** Yuqing Zhu, Chadbourne M. B. Smith, Tarek Jabri, Mufeng Tang, Franz Scherr, Jason N. MacLean

**Affiliations:** 1 Committee on Computational Neuroscience, University of Chicago, Chicago, IL United States of America; 2 Department of Neuroscience, Pomona College, Claremont, CA United States of America; 3 Department of Neurobiology, University of Chicago, Chicago, IL United States of America; 4 Department of Psychology and Neuroscience, Duke University, Durham, NC United States of America; 5 Department of Statistics, University of Chicago, Chicago, IL United States of America; 6 Medical Research Council Brain Network Dynamics Unit, University of Oxford, Oxford, England, UK; 7 Amazon, London, United Kingdom; 8 Institute of Theoretical Computer Science, Graz University of Technology, Graz, Austria; 9 Huawei Technologies Switzerland, Zurich, Switzerland; 10 Flexion Robotics, Zurich, Switzerland; 11 Department of Neurobiology, Harvard Medical School, Boston, MA United States of America

**Keywords:** Spiking neural networks, Recurrent neural networks, Interneurons, Inhibition, Trained network models

## Abstract

The neocortex is composed of spiking neurons interconnected in a sparse, recurrent network. Spiking activity within these networks underlies the computations that transform sensory inputs into appropriate behavioral responses. In this study, we train recurrent spiking neural network (SNN) models constrained by neocortical connectivity statistics and investigate the architectural changes that enable task-relevant, spike-based computations. We employ a binary state change detection task—an experimental paradigm used in animal behavioral studies. Our SNNs consist of interconnected excitatory and inhibitory units with connection probabilities and strengths modeled after the mouse neocortex and maintained throughout training and evaluation. Following training, we find that SNNs selectively modulate firing rates based on the binary input state, and that excitatory and inhibitory connectivity within and between input and recurrent layers adjusts accordingly. Notably, inhibitory neurons in the recurrent layer that positively modulate firing rates in response to one input state strengthen their connections to recurrent units with the opposite modulation. This push-pull connectivity—where excitation and inhibition are dynamically balanced in an opponent fashion—emerges as a key computational strategy and is reminiscent of connectivity observed in primary visual cortex. Using a one-hot output encoding yields identical firing rates to both input states, yet the push-pull inhibitory motif still arises. Importantly, this motif fails to emerge when Dale’s principle is not enforced during training, and task performance also declines.Furthermore, disrupting spike timing by a few milliseconds significantly impairs task performance, highlighting the importance of precise spike time coordination for computation in sparse networks like neocortex. The emergence of push-pull inhibition through task training in spiking models underscores the crucial role of interneurons and structured inhibition in shaping neural dynamics and spike-based information processing.

## Introduction

Neocortical networks use neuronal spikes to perform computations that ultimately drive purposeful animal behavior. While definitions of computation vary, one way to study it is through the input-output transformations that allow animals to generate behavior appropriate to sensory inputs. By training spiking neural networks (SNNs) with recurrent connectivity inspired by the neocortex, we can investigate computational mechanisms by comparing models that successfully perform computations versus those that do not. Here we study how a recurrent spiking network—mirroring key aspects of cortical circuits—can separate, amplify, or suppress sensory inputs and route them to the correct output through structured connectivity changes during training.

We focus on recurrent SNNs in this study because we aim to understand how network models more similar to neocortex can learn to perform even simple computations. By equipping our model with the ability to spike, just as biological neurons do, we allow it to discover solutions that utilize spikes. By equipping our SNN with recurrent connectivity, as observed in neocortex, we allow it to discover solutions that make use of this network structure.

Previously, SNN models have enriched our understanding of the mechanisms underlying neocortical activity. Prior work has explored how network properties such as information capacity (Brunel 2016), dynamic range (Shew et al. 2009), information transmission (Mejias and Longtin 2012), and decodability (Cohen et al. 2020) contribute to cortical function. Notably, asynchronous spiking activity, a hallmark of neocortex, has been shown to enhance coding capacity (Kohn et al. 2016; Lankarany and Prescott 2015). Advances in training SNNs for tasks (Lee et al. 2016; Huh and Sejnowski 2018; Bellec et al. 2020; Zenke and Vogels 2021) enable us to apply similar approaches to task-optimized SNNs.

Trained SNNs employ spike-based computations (Brette 2015; Verzi et al. 2018) while capturing network nonlinearities (Brette and Gerstner 2005). Spikes are fundamental to neocortical information processing. The relative timing of spikes has been demonstrated to carry information about stimulus features in neocortex (deCharms and Merzenich 1996). The discrete and sparse nature of spikes can help mitigate the impact of noisy inputs (Calaim et al. 2022), especially when neuronal units themselves are also leaky (Sharmin et al. 2020). This makes SNNs especially suitable for modeling neural systems operating under realistic, noisy conditions. Individual spikes also have genuine efficacy, as they are used by animals to drive their own behaviors. The earliest stimulus-evoked spikes in mouse primary visual cortex (V1) are preferentially weighted for guiding behavior (Day-Cooney et al. 2022), and mice are capable of performing a visual discrimination task within a narrow time window during which the majority of V1 neurons involved in the task fire either one or no spikes (Resulaj et al. 2018). By focusing on spiking models, we ensure our investigation remains relevant to the computations performed by biological neurons, which rely on the discrete nature of spikes.

Guided by experimental observations, we built SNN models incorporating neocortical structural and dynamic features and trained them to perform a binary report task that mice can perform. Our results reveal the emergence of a push-pull connectivity motif: excitatory connections strengthen between neurons sharing the same stimulus preference, while inhibitory neurons selectively suppress neurons tuned to opposing stimuli. This result strengthens existing experimental hypotheses for why neocortical networks exhibit structured excitatory-inhibitory interactions. Both input layer and recurrent layer connections are an important part of the model’s solution, highlighting how thalamic inputs and recurrent cortical activity work in tandem to process sensory input to guide behaviors.

Furthermore, our study underscores the broad importance of precise spike timing–even though the task did not require rapid outputs–in networks with sparse activity and weak synapses like neocortex. Millisecond-scale disruptions to spike timing impaired the model’s task performance by disrupting its ability to give steady outputs, reinforcing the theory that computations in the neocortex rely on precise spike coordination.

## Results

### Building and training recurrent spiking neural network models

We built and trained several model variations for this study. We begin with reporting the structure and results of our default model and present variations in Sect. 2.6 onwards.

**Fig. 1:**
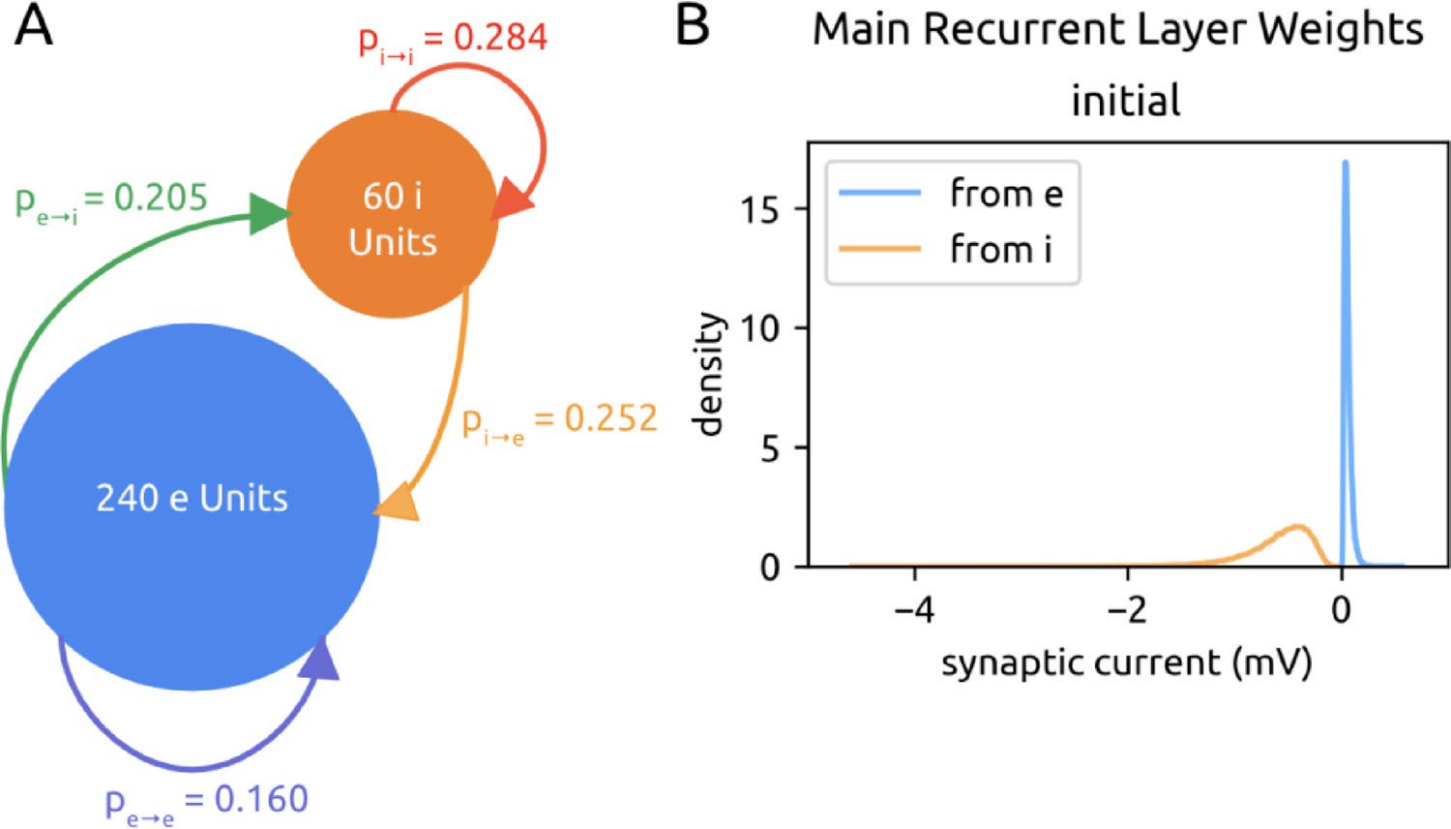
Network architecture. **A**. The main recurrent SNN is made of 240 excitatory (e) units and 60 inhibitory (i) units, connected within and between themselves with probabilities of connectivity taken experimentally from mouse neocortex. **B**. Distribution of initial recurrent e and i weights, pooled across all experiments

Our SNN models were composed of 240 excitatory (e) and 100 inhibitory (i) adaptive leaky integrate-and-fire (ALIF) neuronal units. Units are recurrently connected via current-based synapses, with probabilities of connection within and between e and i populations drawn from mouse visual cortex (Billeh et al. 2020; Jabri and MacLean 2022) (Fig. 1A). Initial e synaptic weights followed a long-tailed distribution (Song et al. 2005; Bojanek & Zhu et al. 2020). Initial i weights followed a similar distribution, only negative and 10x stronger (Litwin-Kumar and Doiron 2012) (Fig. 1B).

**Fig. 2:**
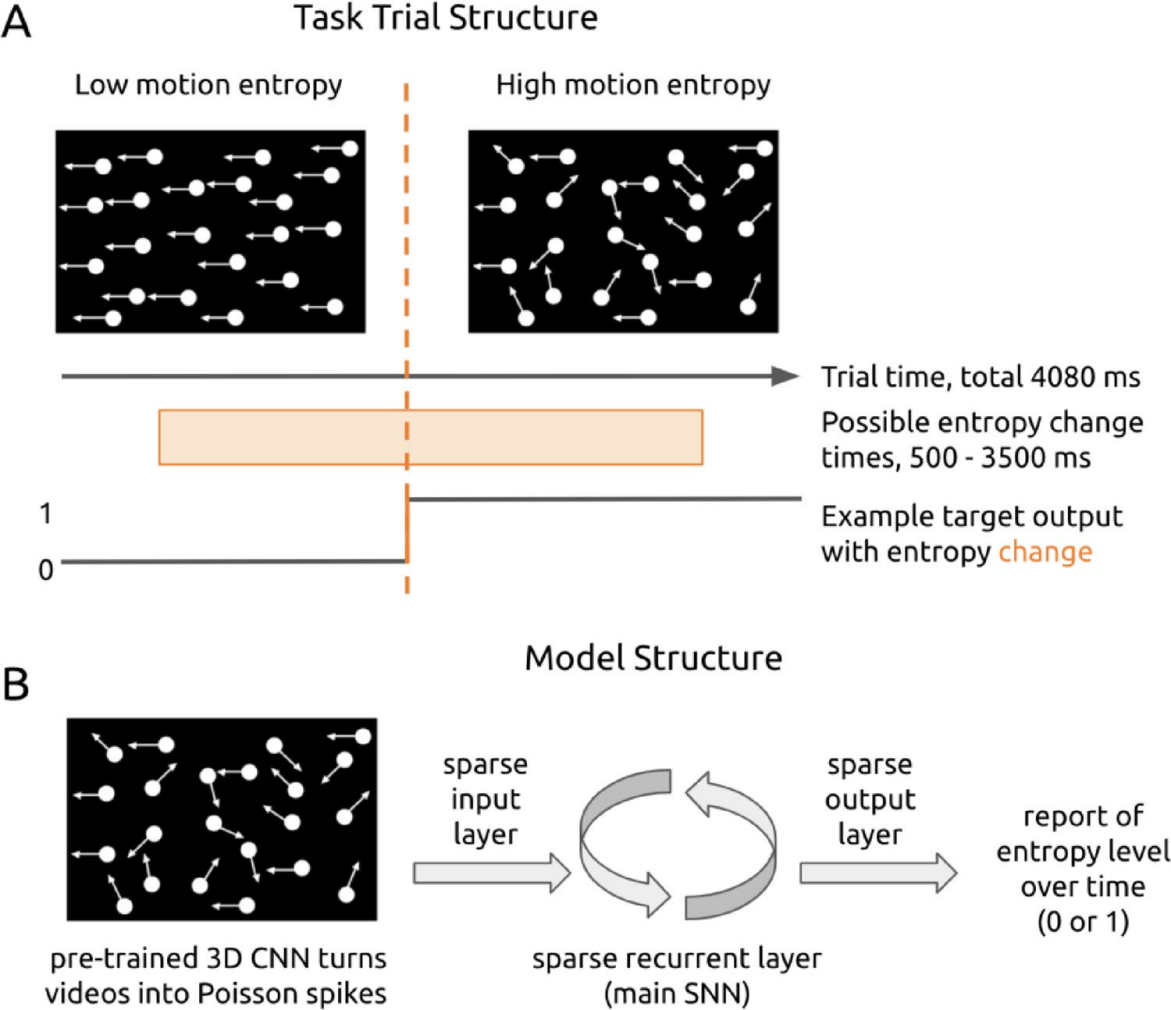
Task and model structure. **A**. In each trial, the model is presented with a 4080-ms video of drifting dots. Dots move at a high (15% same direction) or low (100% same direction) motion entropy level. The model reports the motion entropy level over time. In half of all trials, the entropy level changes at a random time between 500 to 3500 ms. **B**. The SNN receives video input from 16 input channels in the form of Poisson spikes. These 16 input channels convey the activation of a velocity-trained 3D CNN (see Methods and Fig. 13) in response to video input. The output of the SNN is a vector of 0s and 1s over time to signal the motion entropy level at each ms

Individual connections within and between e and i populations were permitted to change during training, yet overall sparse probabilities of connectivity were maintained according to the DEEP R algorithm (Bellec et al. 2018). Positivity and negativity of all connections, i.e. the excitatory or inhibitory identity of each neuron, was maintained throughout training consistent with Dale’s law (Kandel 1957). Models were trained on a visual motion entropy change detection task, consistent with tasks that mice are able to learn (Douglas et al. 2006; Kirkels et al. 2018; Marques et al. 2018) (Fig. 2A). The model was presented with videos of drifting dots moving at two levels of motion entropy. In the high entropy case, 15% of dots have the same direction of motion; in the low entropy case, 100% of dots have the same direction of motion. Half of all trials had a change in motion entropy which occurred at a random time between 500 and 3500 ms within the 4080 ms total duration of the trial.

**Fig. 3:**
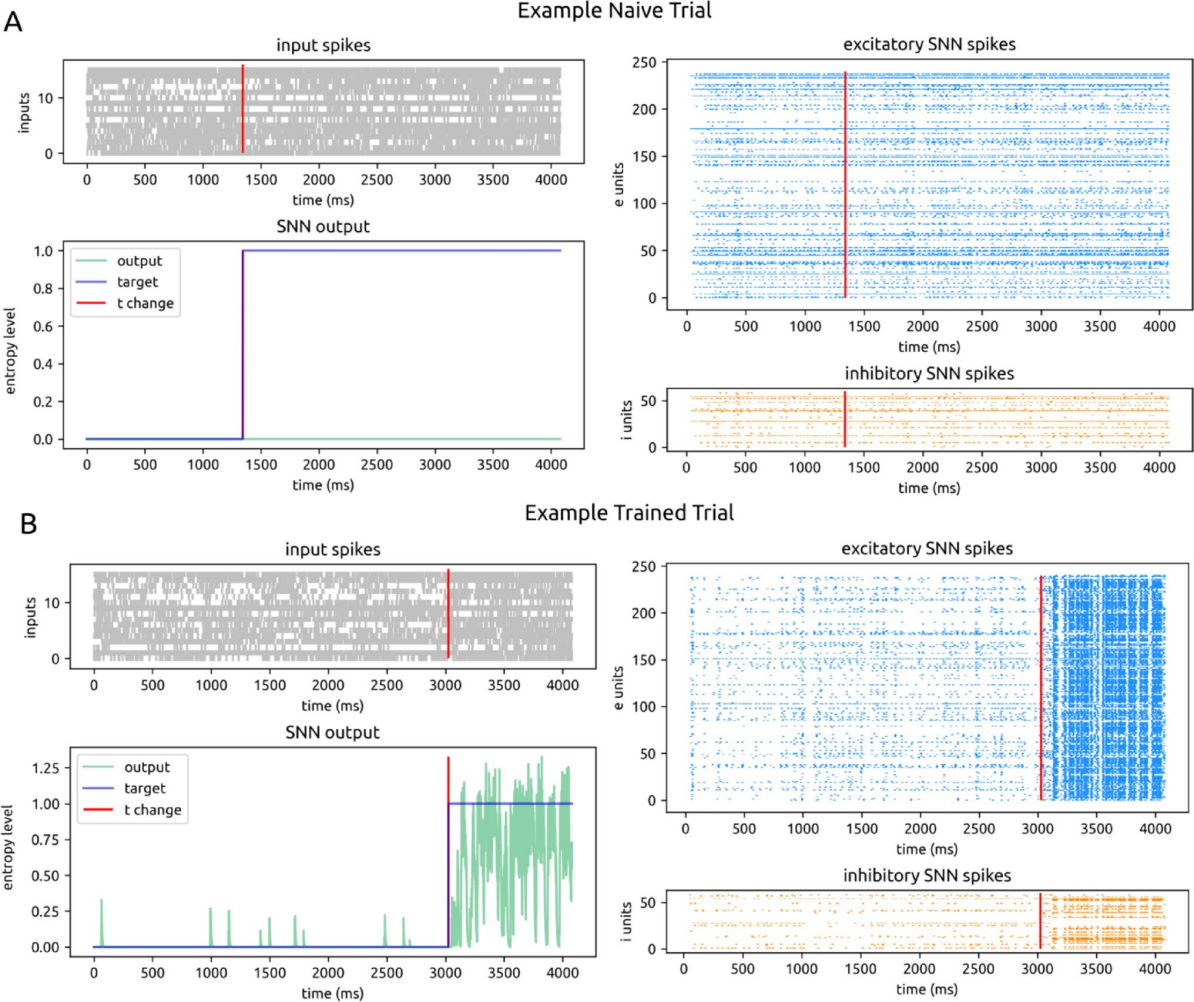
Example model activity. **A**. An example of an untrained trial in which a motion entropy change occurred. All plots are displayed over time (4080 ms trial duration) on the x-axis. The red vertical line shows the time of motion entropy change. Top left: spikes from 16 input channels. Bottom left: model output for motion entropy level in green; true target motion entropy level in violet. Top right: spikes from 240 excitatory units in the main recurrent SNN. Bottom right: spikes from 60 inhibitory units in the main recurrent SNN. **B**. Same as Fig. 3A for an example trained trial

To enable the SNN to interpret input videos, a pre-trained 3D convolutional neural network (CNN) was used to convert videos into spike sequences (Fig. 3, Fig. 13). The output activations of the 16 units in the CNN’s last layer are interpreted as firing rates to generate Poisson spikes (see Methods Sect. 4.13). These 16 units were the SNN’s input channels.

Models had a single output unit to instantaneously report the motion entropy level at each ms time step of the trial (Fig. 2B). The target output sequence is composed of 0’s and/or 1’s; the number assigned to each motion entropy level is randomly swapped in different experiments. The total deviation (as measured by the MSE) between the model’s motion entropy prediction and the target motion entropy for all 4080 ms of a trial is the task loss for that trial.

In addition to task loss, we define a rate loss that is used to encourage naturalistic sparse spiking dynamics (Koulakov et al. 2009; Roxin et al. 2011). A target spike rate was specified (20 Hz, or 0.020 spikes/ms), and the deviation (MSE) between it and the recurrent SNN’s average spike rate is the rate loss for a given trial. The rate loss could be added to the task loss and networks could be optimized on both metrics (Zhu et al. 2020).

**Fig. 4:**
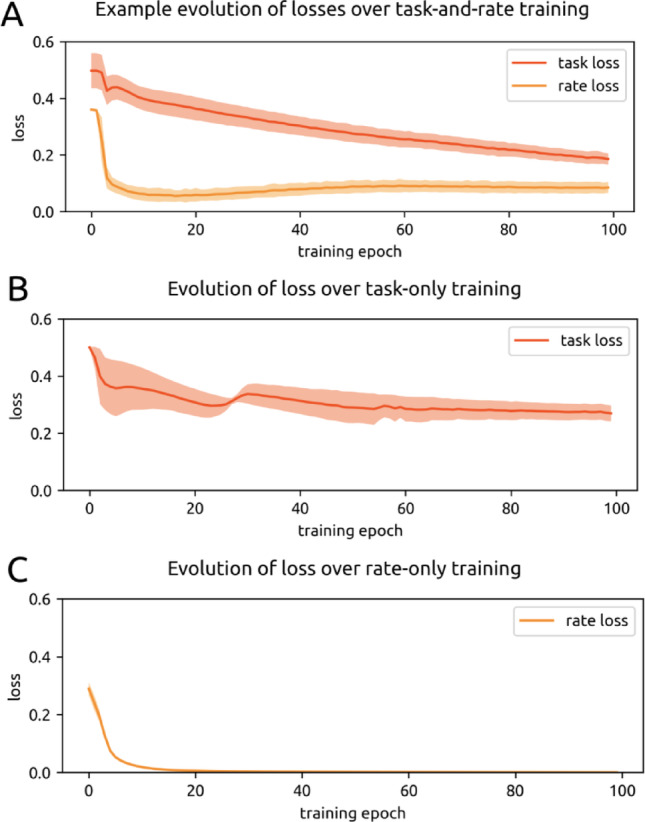
Loss. **A**. Losses over training for an example experiment in which the model was trained to minimize both task (red) and rate (orange) loss. Solid lines depict the mean loss across timepoints and trials in each epoch, and shaded areas depict +/- one standard deviation. **B**. Loss over training for all experiments in which the model was trained to minimize only task loss. **C**. Same as Fig. 4A for only rate loss training

Networks were trained in three ways: to minimize task loss, to minimize rate loss, and to minimize both– which we define as dual loss (Fig. 4). We focus on dual loss; results from training on only rate and only task loss are reported as controls / points of contrast. All models were trained for 10,000 batch updates (100 epochs of 100 batches each), where each batch consisted of 30 distinct trials.

As all networks were initialized with the same protocol regardless of the type of optimization, the initial starting losses across all types of training (*n* = 77) were similar, with initial task loss at 0.50 ± 0.07 and initial rate loss at 0.39 ± 0.08. Across dual loss training sessions (*n* = 28), models achieved 0.28 ± 0.15 task loss and 0.2 ± 0.2 rate loss after training (Fig. 4A). After training on task alone (*n* = 16), models achieved 0.27 ± 0.02 task loss. Models tended to progressively elevate firing rates during task-only training, with rate loss increasing to 13 ± 10 by the final epoch. After training on rate alone (*n* = 38), models achieved 0.0007 ± 0.0002 rate loss. Thus training on rate alone leads to better rate performance than when training to minimize both rate and task loss, but training on task alone does not lead to much better task performance than when training to minimize both rate and task loss.

Weights of all layers (input, recurrent SNN, and output) were permitted to change during any type of training. We used the Adam optimizer, which adapts the learning rate for every variable over the course of training (Kingma and Ba 2014). SNNs were trained using BPTT with surrogate spike gradients (see Methods Sect. 4.8–4.10, Huh and Sejnowski 2018; Bellec et al. 2020; Zenke and Vogels 2021).

**Fig. 5:**
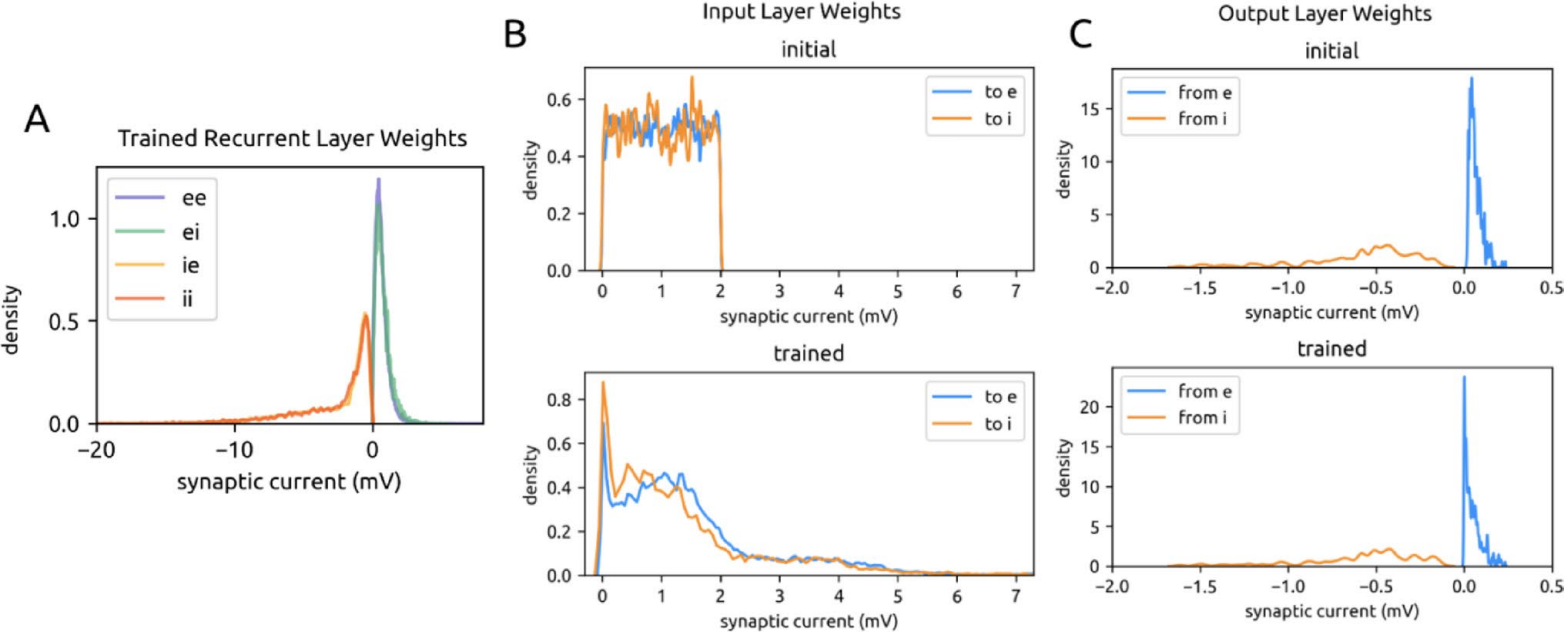
Weight changes during dual training. **A**. Trained weight distributions for the main recurrent SNN pooled across dual training experiments. Connections are separated by e→e, e→i, i→e, and i→i type. **B**. Initial (top) and trained (bottom) input layer weights to main SNN e units (blue) and i units (orange). Input weights were initialized with a uniform distribution [0, 2]. **C**. Same as Fig. 5B for the output layer; in blue are the weights from main SNN e units and in orange are the weights from i units

SNN input and output layers were also sparsely connected, matching the statistics of the recurrent layer (Fig. 5). We further specified that no recurrent layer units which received input could directly project to output. By stipulating these architectural and training details, we ensured that weight changes which supported task learning preferentially took place in the recurrent layer.

### After Training, models demonstrate significant synaptic weight remodeling in the recurrent layer

Synaptic weights are long tailed in neocortex and all models were initialized accordingly. This distribution is not enforced during training, yet we observed that the shape is preserved (Fig. 5A). Weights in the recurrent layer became approximately 10x stronger.

Separating the recurrent network by excitatory and inhibitory units, weights change as follows (Table 1):

**Table 1:** Weight changes through dual training

connection type	Initial (mV)	Trained (mV)
Input layer		
in→e	0.3 ± 0.6	0.4 ± 1.0
in→i	0.3 ± 0.6	0.4 ± 1.0
Recurrent layer		
e→e	0.01 ± 0.03	0.1 ± 0.4
e→i	0.01 ± 0.03	0.1 ± 0.3
i→e	-0.2 ± 0.3	-1 ± 2
i→i	-0.2 ± 0.3	-1 ± 2
Output layer		
e→out	0.01 ± 0.03	0.01 ± 0.02
i→out	-0.2 ± 0.3	-0.2 ± 0.3

Recurrent layer weight changes for rate-only-training and task-only-training are similar (see Methods Sect. 4.19, Tables 2, 3).

**Table 2:** Weight changes through rate training

connection type	initial (mV)	trained (mV)
Input layer		
in→e	0.2 ± 0.5	0.3 ± 0.8
in→i	0.2 ± 0.5	0.2 ± 0.6
Recurrent layer		
e→e	0.01 ± 0.03	0.1 ± 0.4
e→i	0.01 ± 0.03	0.2 ± 0.4
i→e	-0.2 ± 0.3	-0.5 ± 1.7
i→i	-0.2 ± 0.3	-0.4 ± 1.5
Output layer		
e→out	0.01 ± 0.03	0.01 ± 0.03
i→out	-0.2 ± 0.3	-0.2 ± 0.3

**Table 3:** Weight changes through task training

Connection type	Initial (mV)	Trained (mV)
Input layer		
in→e	0.3 ± 0.6	0.4 ± 0.7
in→i	0.3 ± 0.5	0.3 ± 0.6
Recurrent layer		
e→e	0.01 ± 0.03	0.1 ± 0.4
e→i	0.01 ± 0.03	0.1 ± 0.3
i→e	-0.2 ± 0.3	-0.7 ± 1.9
i→i	-0.2 ± 0.3	-0.7 ± 1.9
Output layer		
e→out	0.01 ± 0.03	0.01 ± 0.02
i→out	-0.1 ± 0.3	-0.1 ± 0.3

Input layer weights were initialized with a uniform distribution but became more long-tailed through training (5B). The functional role of input layer structural changes is addressed in Sect. 2.4. The output layer’s weights were initialized with the same distribution as the recurrent layer’s weights, and this distribution is largely preserved through training (5 C).

We next investigated whether the recurrent connections with the strongest initial weights still had the strongest weights after training. We identified the recurrent layer connections which had the top decile of initial absolute weights. After dual training, only 3.9 ± 1.4% of those connections remained in the top decile of weights. When we broadened the set of connections to the starting top quartile of weights, only 10 ± 3% of those connections remained in the top quartile after dual training. Therefore, strong initial weights did not determine strong final weights in the recurrent network.

### **Models modulate firing rates to solve the task**

**Fig. 6:**
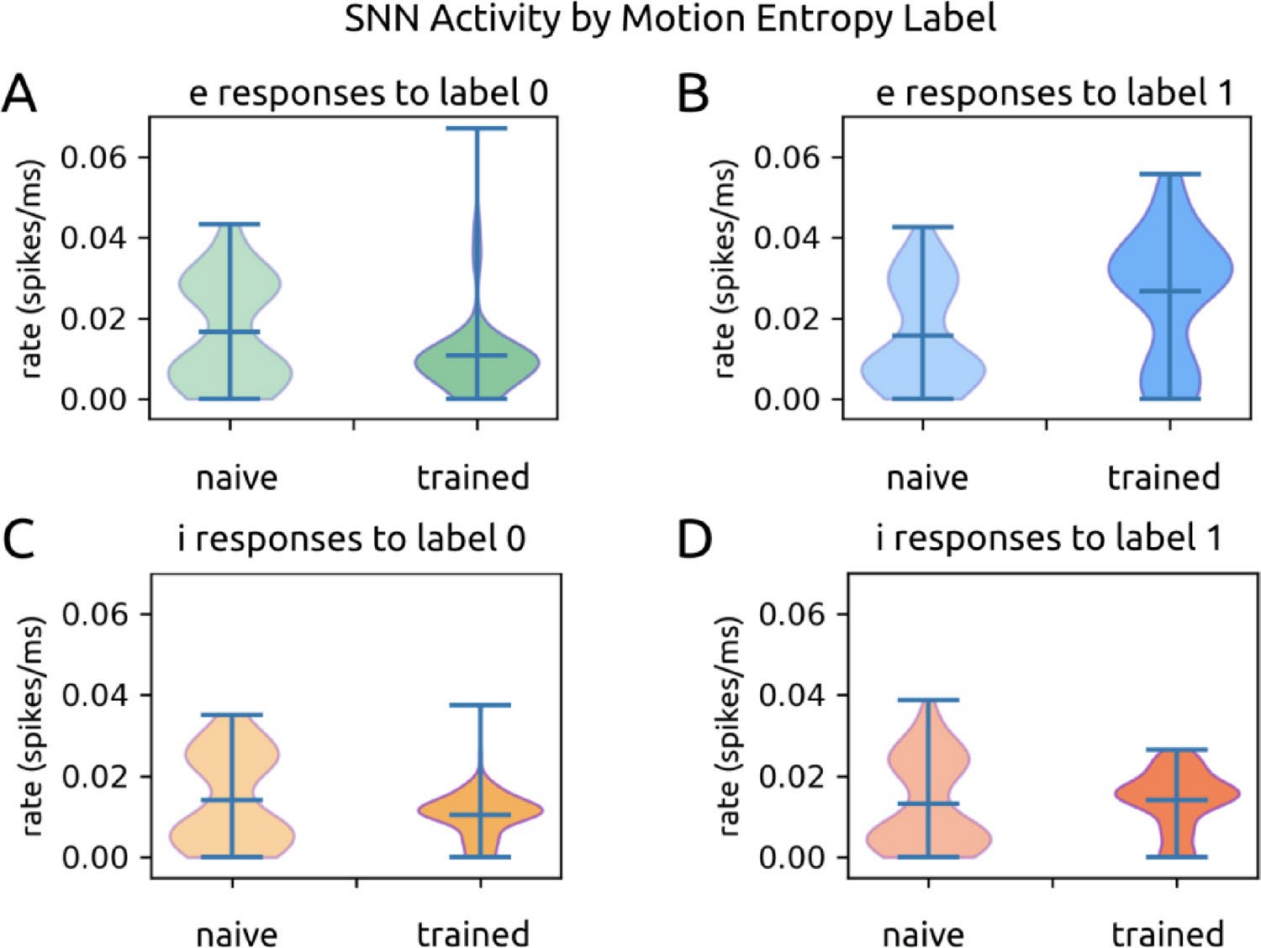
SNN activity in response to motion entropy levels. **A**. Excitatory units’ firing rates in response to motion entropy level ‘0’ in the initial (left) and trained (right) states. Rates are pooled over all dual training sessions. **B**. same as Fig. 6B for entropy level ‘1’. **C**. Same as Fig. 6A for inhibitory units. **D**. Same as Fig. 6C for entropy level ‘1’

We found that models increasingly modulated their firing rates according to the output label associated with each motion entropy level over the course of training. Models increased firing rates in response to the motion entropy labeled ‘1’ and decreased firing rates to the motion entropy labeled ‘0’ (Figs. 3B and 6). It did not matter whether ‘1’ was attached to low or high motion entropy; networks trained with swapped labels exhibited the same behavior, in which the ‘1’ label became associated with elevated firing.

In the untrained state (*n* = 28), e units responded with similar firing rates to both labels (label ‘0’: 0.017 ± 0.013 spikes/ms; label ‘1’: 0.016 ± 0.013 spikes/ms; *p* = 0.376) (Fig. 6A; B). I units also responded similarly to the two labels (label ‘0’: 0.014 ± 0.011 spikes/ms; label ‘1’: 0.013 ± 0.011 spikes/ms; *p* = 0.040) (Fig. 6C; D).

In the dual-trained state, e units responded with lower firing to state ‘0’ (0.011 ± 0.010 spikes/ms) and higher firing to state ‘1’ (0.027 ± 0.016 spikes/ms, *p* = 6.661 · 10^− 16^ ) (Fig. 6A; B), representing a ~ 2.5x increase in firing rates to the ‘1’ state label. To a lesser extent, i units also decreased firing rates for state ‘0’ (0.011 ± 0.006 spikes/ms) and increased firing for state ‘1’ (0.014 ± 0.007, *p* = 1.147 · 10^− 13^) (Fig. 6C; D).

We next investigated how the models’ connectivity changed over the course of training to yield this reliable difference in response to the two labels. We find that changes in i connections of both the input and recurrent layers support this difference.

### Input channels strengthen connections to E or I recurrent layer units according to motion entropy modulation

**Fig. 7:**
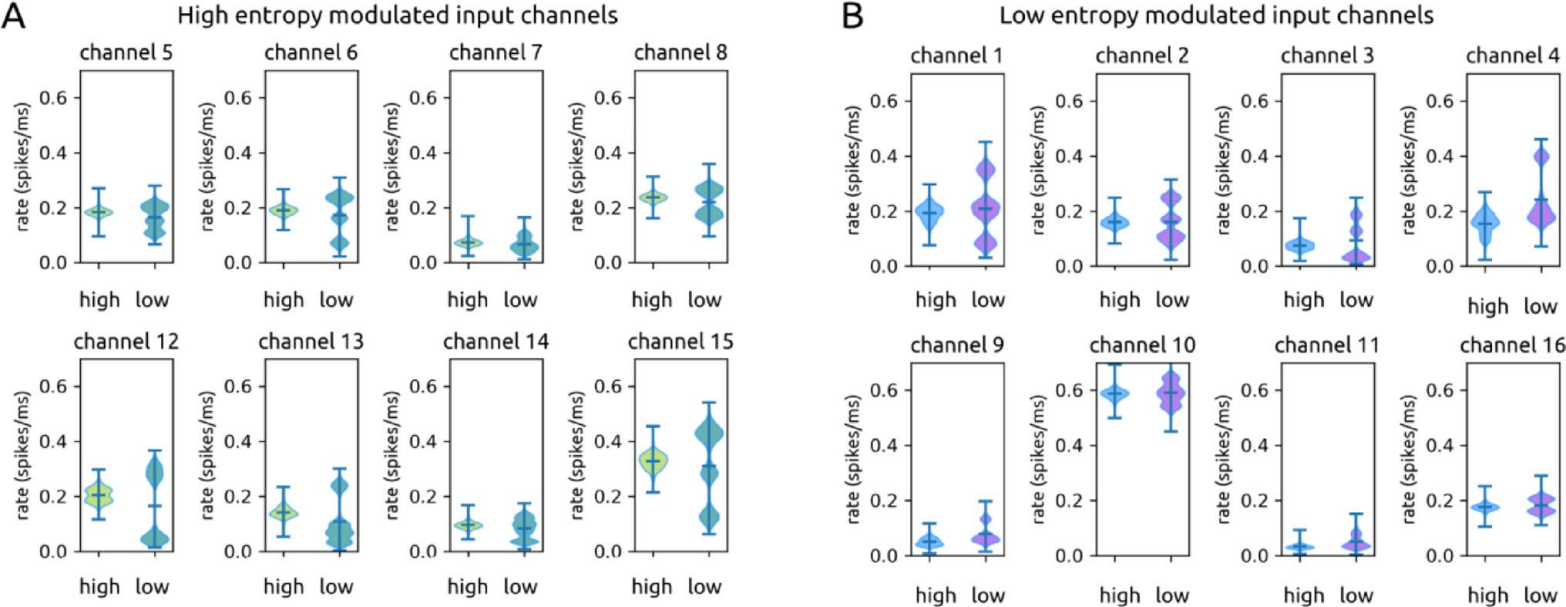
Motion entropy modulation of input channels. **A**. Violin plots of input channels’ firing rates in response to high (left violins) and low (right violins) motion entropy levels. The top, middle, and bottom bars of each violin are the maximum, mean, and minimum respectively. The width of each curve corresponds to the frequency of data points. Figure 7A shows the 8 channels that have higher mean firing rates for high entropy. **B**. Same as Fig. 7A for the 8 channels that have higher mean firing rates for low entropy

All 16 input channels had different mean firing rates in response to the two motion entropy states (all *p* ≈ 0.0), with a difference of 0.021 spikes/ms on average (details in Methods Sect. 4.14). Notably, half of the input channels responded with elevated mean firing during periods of high motion entropy (response to high entropy: 0.18 ± 0.08 spikes/ms; response to low entropy 0.16 ± 0.11 spikes/ms; *p* ≈ 0.0) (Fig. 7A). The other half responded with elevated rates to low motion entropy (response to high entropy: 0.18 ± 0.17 spikes/ms; response to low entropy: 0.20 ± 0.16 spikes/ms; *p* ≈ 0.0) (Fig. 7B). We refer to these input channels as high-entropy-modulated and low-entropy-modulated input channels respectively.

**Fig. 8:**
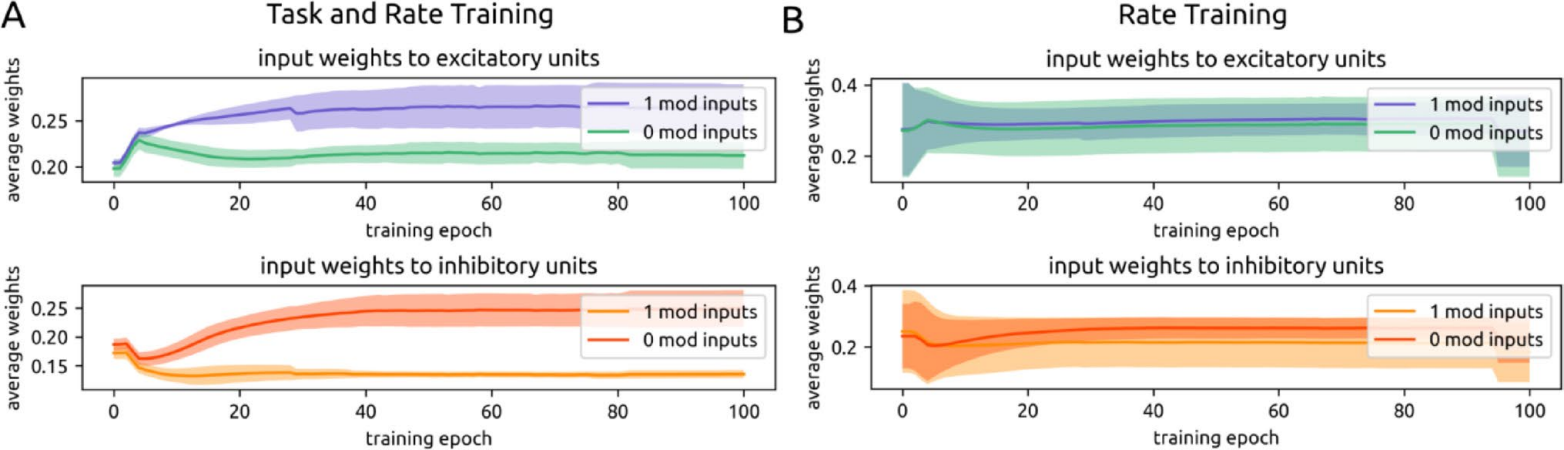
Selective strengthening of input layer weights according to input modulation. **A**. Over the course of dual training, input channels positively modulated by motion entropy 1 develop stronger connections to excitatory units (top), while input channels positively modulated by motion entropy 0 develop stronger connections to inhibitory units (bottom). **B**. This pattern is not observed during only rate training

Each input channel synapses onto subpopulations of units in the recurrent layer, including both e and i units. Prior to training, both high-entropy-modulated and low-entropy-modulated input channels had similarly weighted connections onto both e (high entropy: 0.198 ± 0.009; low entropy: 0.204 ± 0.004) and i units (high entropy: 0.187 ± 0.010; low entropy: 0.172 ± 0.011) in the recurrent layer. Over the course of training, the two groups of input channels diverged in the strength of their synapses onto e and i recurrent layer units (Fig. 8A).

When high motion entropy is labeled as ‘0’ and low motion entropy is labeled as ‘1’ during training, high-entropy-modulated input channels increase weights onto i units (0.25 ± 0.03) while decreasing weights onto e units (0.212 ± 0.014). On the other hand, low-entropy-modulated input channels develop stronger weights onto e units (0.26 ± 0.03) and weaker weights to i units (0.135 ± 0.007). In this way, when a high entropy input is presented, the network becomes inhibition dominated, leading to lower spike rates and a report of the lower output label (‘0’). Conversely, when a low motion entropy input is presented, the network becomes driven by excitation and reports the higher output value (‘1’).

If the labels are swapped, the opposite changes in connectivity occurred. High-entropy-modulated input channels developed stronger weights onto e units and low-entropy-modulated input channels developed stronger weights onto i units. Thus, over the course of training, input channels that preferentially responded to a particular motion entropy level became more strongly connected to either e or i units, resulting in either high or low firing states to match the desired output label.

This result can be summarized as a ratio of 1-modulated input weights / 0-modulated input weights onto i and e units in the recurrent layer. Again, 0 can refer to either low or high motion entropy; what is important is which entropy level is given the ‘0’ label for that experiment. For i units, this ratio begins at 0.921 and becomes 0.543 after training. This means that through training, i units receive approximately twice as much drive from input channels modulated by the ‘0’ entropy state. For e units, this ratio begins at 1.031 and becomes 1.239 after training. Thus following training, e units receive moderately more input drive from ‘1’-modulated input channels.

In versions of the model trained only on rate, we do not observe this separation of inputs to e or i recurrent layer units (Fig. 8B). For i units, the average ratio of ‘1’-modulated input weights over ‘0’-modulated input weights begins at 0.970 and becomes 0.821. For e units this ratio begins at 1.010 and becomes 1.054. This is expected, as there is no imperative to drive inhibition or excitation more strongly in order to report ‘1’ or ‘0’ when optimizing for rate loss alone.

The input layer is not the only part of the model that changes to enable rate modulation. Recurrent layer connections play a key role in amplifying this behavior.

### Recurrent I units strengthen connections to recurrent layer units of opposite modulation

Similar to input channels, recurrent layer units also modulated firing rates to one or the other motion entropy label. We define separate populations of ‘0’ or ‘1’ modulated units according to the label for which they positively modulated firing rates at the end of training.

In the dual-trained SNN, the majority of e units respond with elevated mean firing to label ‘1’ (194 ± 60 out of 240 total e units). A smaller proportion (27 ± 16 e units) responded with elevated firing to label ‘0’. Similar numbers of i units respond with elevated firing to ‘1’-labeled (22 ± 8 out of 60 total i units) as to ‘0’-labeled (21 ± 8 i units) entropy input.

**Fig. 9:**
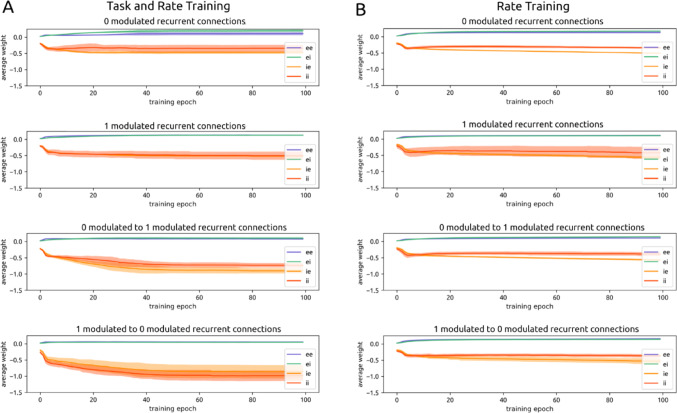
Selective strengthening of recurrent cross-modulation inhibition. **A**. The average strength of recurrent layer ee, ei, i.e., and ii connections, plotted over dual training epochs. Each plot highlights the connections between pairs of units of a particular modulation pattern. From top to bottom: connections between units that are both positively modulated by input ‘0’, connections between units that are both positively modulated by input ‘1’, connections from units positively modulated by ‘0’ to units positively modulated by ‘1’, connections from units positively modulated by ‘1’ to units positively modulated by ‘0’. Over the course of dual training, inhibitory recurrent layer units develop the strongest connections to recurrent layer units of the opposite modulation pattern (i.e. and ii lines in the bottom two plots). **B**. This pattern is not observed when models are trained on only rate

We plotted the mean weights within and between ‘0’- and ‘1’-modulated populations for e→e, e→i, i→e, and i→i connections over the course of dual training (Fig. 9). Plots track retroactively how these units’ weights evolved over training in conjunction with the emergence of positive rate modulation. We found that differences arise in the connectivity between recurrent layer units according to their label preference. In particular, recurrent i units send the strongest connections to units of the opposite modulation (Fig. 9A).

During training, recurrent e weights increase more within the same modulation pattern (approximately 5x relative to initial state) than across (approximately 3x relative to initial state). E weights onto i units increase more within the same modulation pattern (approximately 5x) than across (approximately 3x). In contrast to e units, during training, i weights onto e units increase moderately within the same modulation pattern (approximately 2x) and increase more strongly across modulation (approximately 4x). During training, i units moderately increase weights to other i units of the same modulation pattern (approximately 2x) and more strongly increase weights to other i units of the opposite modulation pattern (approximately 3x). The precise numerical values of the synaptic weights for initial and trained networks are reported in Methods Sect. 4.18 (Table 4).

Thus, over the course of training, e units increase weights to other units of the same modulation pattern, while i units increase weights to units of the opposite pattern, precisely illustrating push-pull connectivity. Two-sample Kolmogorov-Smirnov (KS) testing confirms that the trained weight distributions are different for within- and across-modulation e connections (*p* = 3.205 · 10^− 7^ comparing e→e within- vs. across-modulation; *p* = 3.847 · 10^− 10^ comparing e→i within- vs. across-modulation). This is also true for within- and across-modulation i connections (*p* = 6.323 · 10^− 14^ comparing i→i within- vs. across-modulation; *p* = 5.409 · 10^− 11^ comparing i→e within- vs. across-modulation).

This set of results can be summarized as a ratio: mean weight across-modulation / mean weight within-modulation. For i connections prior to training, this ratio is 1.06 ± 0.15. After training, this ratio increases to 2.0 ± 0.7. Thus i units in trained networks send connections that are approximately twice as strong to opposite-modulated recurrent layer units compared to same-modulated recurrent layer units. For e connections, this ratio is 1.00 ± 0.10 in the initial state and 0.6 ± 0.3 in the trained state. Thus e units exhibit the precise opposite pattern of connectivity as i units after training: they come to send connections that are twice as strong to *same-*modulated recurrent layer units compared to opposite-modulated recurrent layer units.

When networks are trained on rate alone (*n* = 38), this pattern of stronger cross-modulation inhibition is not observed (Fig. 9B). The average ratio of i weights across-modulation / within-modulation is 1.035 for initial networks and 1.027 for trained networks. For e weights, the ratio is 0.996 for initial networks and 1.051 for trained networks. The proximity of this ratio to 1 informs us that the strength of across-modulation vs. within-modulation connections are approximately equal in strength for the rate-trained network.

### Cross-Modulation Inhibition arises despite weaker initial Inhibition

**Fig. 10:**
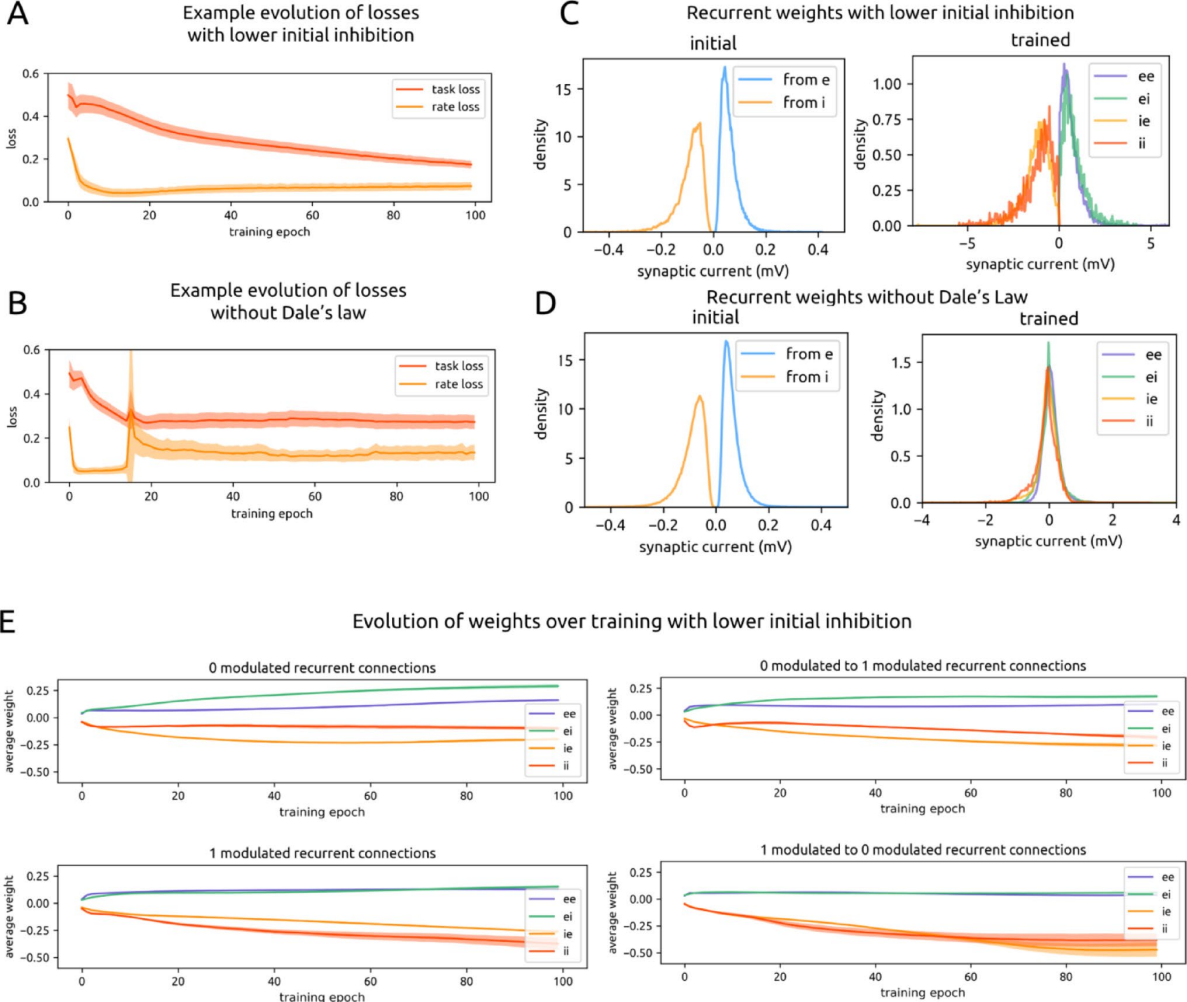
Relaxing constraints on inhibition. **A**. Task and rate loss over the course of training for an example experiment in which the model was initialized with lower inhibitory weights (1.5x as strong as excitatory weights, compared to 10x previously). **B**. Same as Fig. 10A for a model without Dale’s law constraints. **C**. Initial (left) and trained (right) weight distributions for all models with lower initial inhibition. Note the ~ 10x difference in x axis scale. **D**. Same as Fig. 10C for all models trained without Dale’s law constraints. Weights are grouped (i.e. into ee, ei, i.e., and ii) according to the initial identity of the connection. Only nonzero weights are included in the distribution, as is the case with all other weight distribution plots. **E**. Same as Fig. 9A for all models with lower initial inhibition

Through their strong cross-modulation connectivity, i units enable the appropriate modulation of high and low recurrent firing rates in response to ‘1’ and ‘0’ labeled motion entropy states. However, i weights start 10x stronger than excitatory weights; this ratio has been contested, and it is possible that this initialization predisposes the network to reach a particular solution. We next initialized networks (*n* = 21) with recurrent i weights that were only slightly (1.5x) stronger than e weights (Fig. 10C).

Following successful training on task and rate loss (Fig. 10A) in the same manner as before, we found that rate modulation according to motion entropy label persisted (‘1’ mean firing rate: 0.030 ± 0.004 spikes/ms for e units and 0.021 ± 0.005 spikes/ms for i units; ‘0’ mean firing rate: 0.013 ± 0.002 spikes/ms for e units and 0.016 ± 0.002 spikes/ms for i units).

Trained weight distributions (Fig. 10C) differed from when i units were initialized to be 10x stronger. Trained e weights were stronger than before (trained e→e: 0.6 ± 0.6 now, previously 0.1 ± 0.4; trained e→i: 0.8 ± 0.6 now, previously 0.1 ± 0.3). However, trained i weights did not increase as much; they approximately preserved the 1.5x magnitude difference from e weights with which they were initialized (trained i→e: -1.2 ± 0.7 now, previously − 1 ± 2; trained i→i: -1.2 ± 0.9 now, previously − 1 ± 2).

When we examined the evolution of recurrent connectivity over the course of dual training, we found that inhibitory cross-modulation persisted (Fig. 10E). The ratio of mean i weights between oppositely-modulated units over similarly-modulated units was 1.121 in the initial state and 2.002 after training. This reflects the two-fold strengthening of i weights across modulation type versus within modulation type, as seen previously when i weights were initialized to be stronger. For e weights, this ratio was 0.918 in the initial state and 0.466 in the trained state, reflecting that e weights once again become stronger between units of the same modulation type. Thus, despite changes in the overall weight distributions upon initialization and in the trained state, the pattern of stronger cross-modulation inhibition and stronger within-modulation excitation still arose as a task solution.

### Adherence to dale’s law is required

We next investigated the extent to which this pattern of cross-modulation inhibition is preserved in models when, in addition to weakening of initial i weights, Dale’s Law was removed as a constraint during training (*n* = 19). In particular, e units could develop negative outgoing weights, and i units could develop positive outgoing weights. Under these further relaxed conditions, would cross-modulation inhibition still emerge as a preferred task solution?

Following dual training in the same manner as before (Fig. 10B), weight distributions were no longer meaningfully separated into e or i according to the identity of the presynaptic unit (Fig. 10D). The magnitudes of positive and negative weighted connections remained comparable to those of the initial state (e: 0.06 ± 0.03 initial to 0.042 ± 0.23 trained; i: -0.09 ± 0.05 initial to -0.1 ± 0.3 trained). The spread of weights within each population, however, increased substantially after training. Previously, under Dale’s law constraints, all weights increased approximately 10x over the course of training (Figs. 1B and 5A; Table 1); the spread scaled appropriately with the overall weight increase.

We quantified the extent of cross-modulation inhibition with the same ratio as before: the mean weight across-modulation divided by the mean weight within-modulation. Because we could no longer track units and their connections according to e and i identity, we report this ratio for the trained model only. For all negative connections, this ratio was 0.842 in the trained state. For all positive connections, this ratio was 0.914 in the trained state. The proximity of both ratios to 1 indicates that, unlike the model which adhered to Dale’s law, the model without Dale’s law did not rely on cross-modulation inhibition and within-modulation excitation to solve the task. We hypothesize that, since each neuron’s activity could now directly drive both downstream excitation and inhibition, the model developed more specific connectivity towards a solution rather than relying on a specific motif. These specific connections between individual units could no longer be captured by summarizing the e and i population groups. It is also notable that the model without Dale’s law performed less well on the task (Fig. 10B compared to Fig. 4A); this may be due to the challenges of needing to develop hyper-specific connections in order to balance the conflicting effects of excitation and inhibition from individual neurons.

### One-Hot encoding yields similar structural solution

The trained behavior of the recurrent model is dependent on the particular output labels: label ‘1’ elicits higher firing rates while label ‘0’ elicits lower firing rates. We next asked whether cross-modulation inhibition depends on having a single output unit and the rate dichotomy that emerges as a result. We thus implemented one-hot encoding with two readout units, where the greater activity of one unit indicates one motion entropy level, and the greater activity of the other indicates the other motion entropy level. We trained our models (*n* = 14) using the same initial parameters otherwise. These one-hot models achieved a comparable level of performance (task loss: 0.28 ± 0.12) as the original models (task loss: 0.28 ± 0.15).

**Fig. 11:**
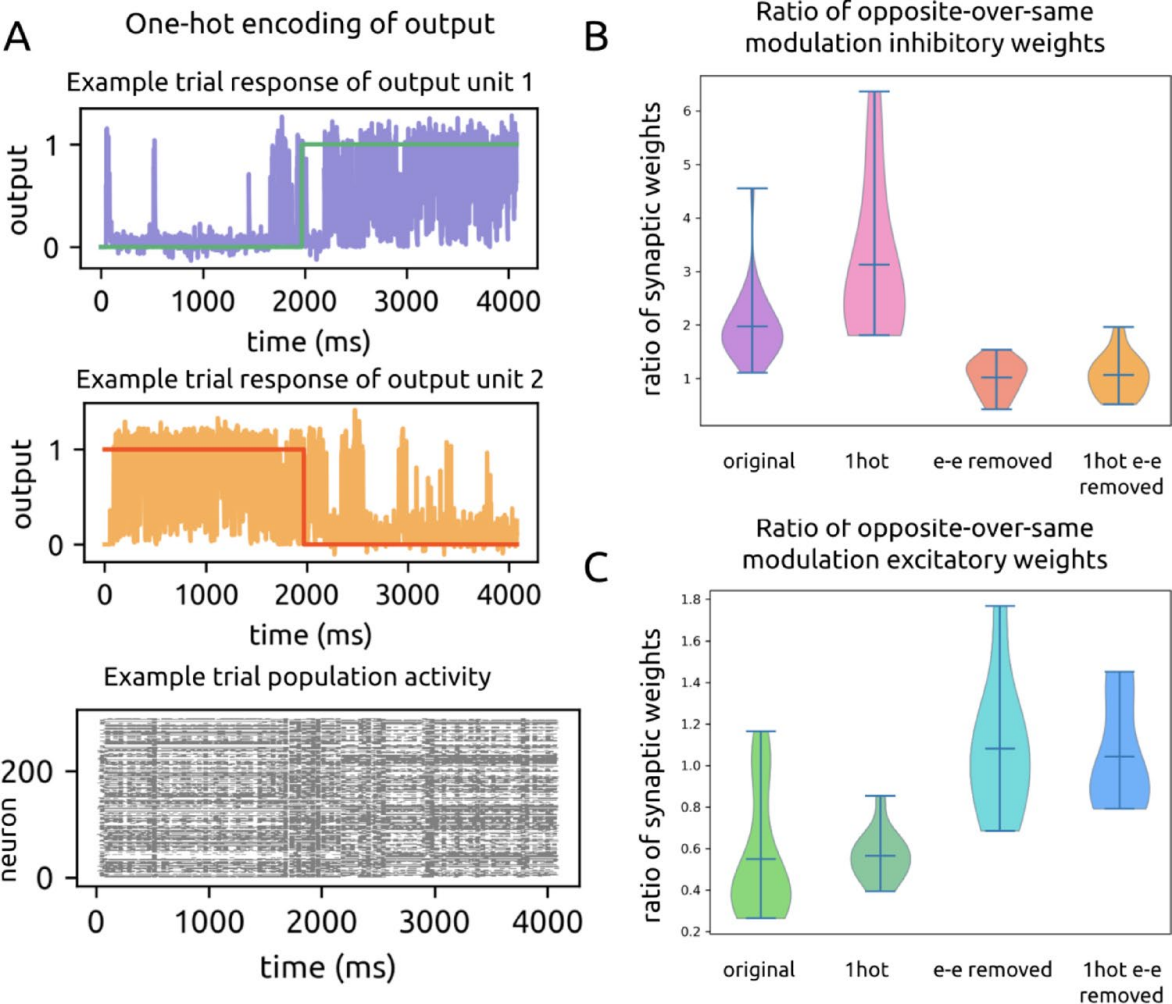
One-hot encoding preserves cross-modulated inhibition, while removal of recurrent excitatory connectivity does not. **A**. Example trained one-hot output activations and raster plot for a trial in which the coherence level changed. **B**. Violin plot of the ratios of recurrent cross-over-within-modulation inhibition from the one-hot model under rate-and-task training. From left to right: original model, one-hot encoding, original model recurrent ee connectivity removed, one-hot encoding recurrent ee connectivity removed. **C**. Violin plot of the ratios of recurrent cross-over-within-modulation excitation from the new experiments with rate-and-task training. From left to right: original model, one-hot encoding, original model with recurrent ee connectivity removed, one-hot model with recurrent ee connectivity removed

After training, whole population firing rates were similar in response to both motion entropy levels (response to level 0: 0.020 ± 0.002 spikes/s; response to level 1: 0194 ± 0.001 spikes/s), with an example trial shown in Fig. 11A. However, individual units still modulated their firing rates more to one entropy level over the other. Across all trained one-hot models, 46.9% of e units and 47.8% of i units were more positively modulated by one level, while the remaining 53.1% of e units and 52.2% of i units were more positively modulated by the other level. Thus we were still able to define units according to their modulation pattern and investigate the extent of cross-modulation connectivity.

We found that with a one-hot encoded output, the model developed even stronger cross-modulation inhibition and within-modulation excitation (Fig. 11B, C). The ratio of mean i weights between oppositely-modulated units over similarly-modulated units was 1.07 ± 0.16 in the initial state and 3.1 ± 1.4 after training. This reflects a three-fold strengthening of i weights across modulation type versus within modulation type, where we found previously a two-fold increase (ratio of 1.06 ± 0.15 initial to 2.0 ± 0.7 trained) with a single readout unit. For e weights, the opposite-modulated to same-modulated ratio was 1.00 ± 0.07 in the initial state and 0.57 ± 0.12 in the trained state, reflecting that e weights become twice as strong between units of the same modulation type. This is a similar ratio of increase as in the single output encoding scheme (ratio of 1.00 ± 0.10 initial to 0.6 ± 0.3 trained).

Our findings show that this architectural solution is effective not only for tasks depending on a single output and clear divisions in whole population activity. It also proves to be a favored approach for tasks requiring different neural populations to produce distinct outputs, as seen with one-hot encoding.

### Recurrent connectivity supports task learning

While binary tasks can be solved by feedforward and non-spiking neural network models, our models are designed based on the recurrent connections and spiking dynamics of neocortex. In the above sections, we report precisely how recurrent spiking models can solve this task through cross-modulation inhibition. In the following sections, we verify that recurrent connectivity and spiking activity are indeed crucial to the learned behavior of our models.

Inspired by recent work (Koren et al. 2024), we removed all e→e connections in the main recurrent SNN with default network initialization, and we prevented them from forming during dual training (*n* = 18). Inputs to the recurrent SNN are untouched, and connections from e units in the recurrent SNN to the output are also untouched. For example, if re1 and re2 are both e units in the main recurrent network, then both re1→re2 and re2→re1 are disallowed connections. In other words, the weights between re1 and re2 must remain at 0 throughout training. Even without recurrent e→e connections, e neurons can still participate in the input–output transformation by receiving input from either recurrent i units or any of the 16 input channels, and by sending output to recurrent i units or directly to the output.

We found that these models performed significantly worse on the task than those which contained recurrent e connectivity (previous task loss: 0.28 ± 0.15, new task loss = 0.33 ± 0.15, *p* = 9.005 · 10^− 31^).

Furthermore, networks that lacked recurrent e→e connectivity did not develop stronger cross-modulated i connections or stronger within-modulated e connections during training. The ratio of mean i weights between oppositely-modulated units over similarly-modulated units was 1.06 ± 0.09 in the initial state and 1.0 ± 0.3 after training. The ratio of mean e weights between oppositely-modulated units over similarly-modulated units was 1.01 ± 0.05 in the initial state and 1.1 ± 0.3 in the trained state (Fig. 11B, C), although now this ratio only represents recurrent e→i connections. These numbers demonstrate that both within- and cross-modulation connection strengths were similar for e and i units and remained largely unchanged during training.

We repeated the previous experiment using one-hot models (*n* = 23) and found that removing recurrent e→e connectivity also resulted in poorer performance (previous one-hot task loss: 0.28 ± 0.12, new one-hot task loss: 0.37 ± 0.15, *p* = 1.378 · 10^− 75^) and a lack of both cross-modulated inhibition and within-modulation excitation. The ratio of mean i weights between oppositely-modulated units over similarly-modulated units was 1.04 ± 0.15 in the initial state and 1.1 ± 0.4 after training. The ratio for e weights was 0.95 ± 0.10 in the initial state and 1.0 ± 0.3 in the trained state (Fig. 11B, C). Once again, across- and within-modulation connection strengths were similar and remained unchanged during training.

Together, these results underscore the combined strategic importance of both cross-modulation inhibition and within-modulation excitation for succeeding at this task, since their absence results in poorer task performance.

### Precise Spike timing supports task solution

To assess the importance of precise spiking for this task, we jittered spike times of all trials in the final epoch for the original, default models (*n* = 17, 51,000 total trials). We measured subsequent output performance using the original output layer weights.

**Fig. 12:**
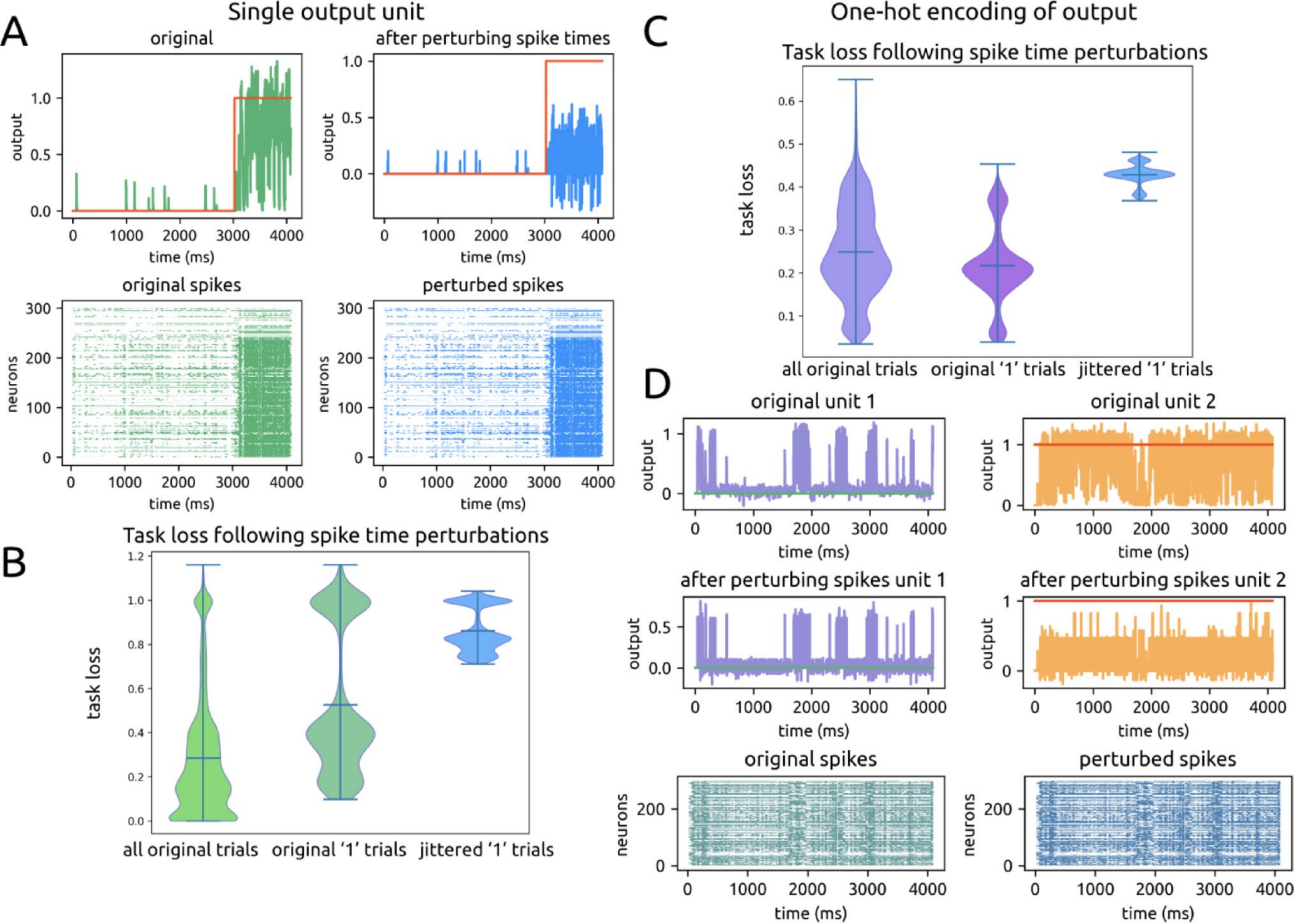
Perturbation of spike times impairs task performance. **A**. Example original spike raster and jittered spike raster for a single trial with corresponding outputs. **B**. Violin plot of task performance on all original trials, the subset of no-change ‘1’-labeled trials, and no-change ‘1’-labeled trials with spike times jittered. **C**. Same as Fig. 12B for one-hot encoding models. **D**. Example trial for one-hot encoding, showing the lack of proper elevation of the two output units at opposite times

We randomly jittered each individual spike for each unit within the range of -5 to 5ms, which is a 0.245% change relative to the duration of each trial (4080 ms) and less than the neurons’ time constant (10 ms). Task performance significantly declined (previous task loss: 0.28 ± 0.15, jittered task loss: 0.4 ± 0.3, *p* ≈ 0) while overall spike rates, by definition, were unchanged. An example of the difference in original and jittered responses is shown in Fig. 12A.

Spike jittering led to substantially worse performance for entropy label ‘1’ as opposed to ‘0’, indicating that the original spikes were timed to enable cooperativity to overcome the weakness of individual synapses and produce a sufficiently strong output value. The original task loss on ‘1’ trials is 0.5 ± 0.3, and the jittered loss on this same set of trials is 0.86 ± 0.10 (*p* ≈ 0), which is significantly higher than the task loss on ‘0’ trials (0.03 ± 0.06, *p* ≈ 0) (Fig. 12B). For label ‘0’, coordination of spike times was not as crucial since this label is defined by low firing rates; performance was thus not as negatively impacted.

We repeated the spike jitter experiments using our one-hot model (*n* = 14, 42,000 total trials). We found similar results in that the task performance was worse after spike jittering (original task loss: 0.3 ± 0.1, jittered task loss: 0.43 ± 0.03, *p* ≈ 0) (Fig. 12C). Under one-hot encoding, the labels ‘0’ and ‘1’ no longer carry significance for the strength of the output; rather, the labels signify which of the two output units should be driven more at each point in time. Thus after spike time jittering, the one-hot model struggled to sufficiently drive the two output units at opposite times, an example of which is shown in Fig. 12D. This behavior once again underscores the importance of spike time coordination for the model’s task solution.

## Discussion

### Visual cortical and thalamic connectivity

Through this work, we provide two circuit mechanisms–one based on input and the other based on recurrent connectivity changes–that underlie task learning. Both highlight the critical role of inhibition in refining sensory inputs to guide task outcomes. Over the course of training, small but significant differences in the input statistics are exploited, and the recurrent network develops strong cross-modulation inhibition to further adjust its responses to be appropriate to the input. Moreover, we achieve this result in a recurrent spiking network, making the mechanisms more directly applicable to recurrent networks of spiking neurons in neocortex.

A biological parallel to the emergence of this pattern–in which units positively modulated by one input came to inhibit units of opposite modulation–can be found in the rich literature of push-pull cortical circuitry (Kremkow et al. 2016; Garcia-Junco-Clemente et al. 2017), cross-orientation suppression of V1 neurons (Morrone et al. 1982; Eysel et al. 1990; DeAngelis et al. 1992; among others) and of the role of interneurons in establishing V1 excitatory selectivity to stimulus orientation and direction.

While the relative contributions of thalamic (feedforward) and intracortical (feedback) connections to establishing V1 selectivity is unresolved (Ferster and Miller 2000; Alitto and Dan 2010; Katzner et al. 2011), one theory is that selectivity arises through a combined effect of initially broad selectivity via thalamic lateral geniculate nucleus inputs that is then sharpened through intracortical feedback (Carandini and Ringach 1997). Our models support this theory in that the strict dichotomy between thalamic and intracortical contributions is false. We find that intrinsic modulation patterns from our input channels, which can be interpreted as modeling thalamic inputs, was refined by recurrent connections in our main SNN, which can be interpreted as a model of V1. As task learning progressed, the local recurrent circuitry underwent changes to enhance the amplification of input modulation patterns by strengthening recurrent excitatory connections from like-modulated excitatory units and cross-modulated inhibitory units.

### Computational roles of interneurons

Intracellular data and modeling work suggest that local intracortical feedback takes the form of inhibitory connections from interneurons that have the same–rather than broader–selectivity as excitatory targets (Katzner et al. 2011). By matching excitatory selectivity, interneurons can precisely keep responses to undesired stimuli below the spike threshold. This is supported by the connectivity observed in our trained models, and the mechanisms underlying this behavior are aligned in both neocortex and the model, since the activity of our model neurons is also fundamentally based on thresholding.

A potential candidate responsible for establishing excitatory selectivity in neocortex is parvalbumin (PV) expressing interneurons. PV neurons synapse onto the soma or axon of synaptic partners (Kepecs and Fishell 2014), allowing them to tightly control spiking in postsynaptic neurons. We used a generic inhibitory neuron in our models, but this property of direct control is a feature of our inhibitory model units. Theoretical work suggests that PV neurons stabilize new groups of task-associated excitatory neurons (Bos et al. 2020; Lagzi et al. 2021; Lagzi and Fairhall 2024), which is consistent with experimental work in associative learning (Morrison et al. 2016). However, results in frontal cortex suggest that vasoactive intestinal polypeptide (VIP) expressing interneurons are the direct inhibitor of excitatory pyramidal cells (Garcia-Junco-Clemente et al. 2017).

A natural extension of our current work is then to diversify neuronal subtypes, as each exhibits unique connectivity properties in local circuits which may point to distinct computational roles (Kepecs and Fishell 2014; Cone et al. 2019). We have now begun this work by differentiating interneurons into PV, SST, and additional subtypes according to distinct structural (Billeh et al. 2020) and dynamic properties such as spike thresholds and time constants (Ayyilmaz et al. 2024).

### Impact of training methods on task solutions

Another extension of this work is to train our models with more realistic learning methods. BPTT, which we used in this study, is generally not regarded as the method by which real neocortical circuits learn. It is possible that the choice of training method can impact the final outcomes of the model. Indeed, when we initially trained SNNs with BPTT and without additional constraints, the models learned to behave as reservoirs. This meant that the recurrent layer’s weights did not change; rather, networks solved the task through changing the readout of pre-existing recurrent dynamics. We investigated whether a reservoir was the optimal solution, or whether it was a consequence of BPTT and other solutions could be discovered via alternate methods (Qu et al. 2024).

In that study, we found that when training via an evolutionary algorithm (EA) on a different task, SNNs underwent changes in recurrent layer connections between the initial and final generations. EA is a more agnostic learning method as it (1) explores a larger space of possible model structures, (2) does not require surrogate gradients for spiking, and (3) mimics how neural structures may evolve to better support computations through the process of natural selection. Thus we concluded that the reservoir solution was a consequence of the BPTT training method, and not truly the best solution. With the addition of realistic constraints, the models used in the present study no longer behaved as reservoirs even when trained with BPTT. Similar to training with EA, changes in recurrent connectivity supported task learning. In the future, we plan to combine EA–which mimics the neural changes that take place in populations over evolutionary time–with local learning rules like spike timing dependent plasticity–which mimics the neural changes during an individual’s lifetime–to train our models and study their solutions. In particular, we will examine if cross-modulation inhibition also arises to solve binary tasks.

### Role of Spike time coordination

We believe the importance of spike time coordination for our models’ solution is a consequence of their sparse, weak synapses and sparse spiking, which mimic the dynamics and connectivity of neocortex. It is important to note that for our task, good performance did not require rapid, millisecond response times from the model. The target output is constant for the majority of each trial. Even so, coordination of recurrent spiking is crucial for maintaining a steady output. By jittering the timing of individual spikes, we disrupted the model’s learned ability to combine into a stable, sufficiently strong output. This result suggests that millisecond spike timing and the use of spiking models may be critical for understanding neural computations even if the task itself does not require precisely timed responses but instead relies on stability.

Consistent with our findings, recent work shows that surrogate gradients, the same method we used, enable SNNs to learn spike-timing-based tasks. As in our work, jittering the timing of individual spikes reduced task performance, with greater jitter causing more severe degradation (Yu et al. 2025). Notably, this study also found that uniformly jittering the entire spike sequence of each neuron produced an even larger performance drop. This difference reflects the disruption to spike-time coordination: per-spike jittering preserves some cross-neuronal coordination by chance, whereas per-neuron jittering eliminates it entirely. Together, these results underscore that cross-neuronal spike-time coordination can be critical for task performance.

### Importance of modeling realistic features under realistic conditions

We acknowledge that the task we used in this study is a relatively simple one. We do not doubt that other models, including non-spiking, feedforward models, could solve this task. A non-spiking recurrent network may even develop cross-modulation inhibition as a solution. However, had we used non-spiking models, we would not have observed our result explaining the broad importance of precise spike timing in networks like neocortex.

On the other hand, a spiking feedforward model may also require precise spike timing to solve the task. Yet had we used such a model, we would not have observed the result confirming the computational relevance of recurrent excitatory-inhibitory motifs. This underscores the need to study networks with realistic features if we wish to understand how complex biological networks solve even the simplest tasks.

On a similar note, a linear decoder of the CNN could’ve solved the task, and we could’ve forewent the recurrent SNN altogether. This is similar to how one can decode many features of a visual stimulus from retinal activity alone. Yet the brain stands in the way between retinal activity and behavior. The intervening brain contributes flexibility, planning, and many other cognitive functions. Future work will therefore place increased task demands on our models, including noisy inputs and more than two output choices. In the meanwhile, our present results demonstrate how a recurrent network uses neocortical circuit motifs and precise spike timing to solve even a simple task. We believe that task-trained SNNs exhibit promise for advancing the understanding of neocortical computation, and we look forward to future work that adopts this method for this goal.

## Methods

### Model construction

Each model is built with spiking units which represent individual neurons. Units are connected with one another via weighted, directed connections which represent current-based synapses. The weight of a connection $\:{W}_{ij}^{rec}$ is a numerical value that indicates the strength of the connection from presynaptic unit $\:i$ to postsynaptic unit $\:j$ within the recurrent SNN.

In a network of 300 neuronal units, 240 are excitatory and 60 are inhibitory. They are built this way to maintain the 4:1 e: i ratio, which is a ratio observed in neocortex. All synaptic connections originating from e units have positive weight values, and all from i units have negative weight values. Positivity and negativity of each connection is maintained during training, although values may change (Zhu et al. 2020).

### Input

Input is delivered in the form of spike sequences onto a subset of the main SNN units. Input spikes are also weighted (e.g. $\:{W}_{i}^{input}$ is the input weight to SNN unit $\:i$), and the weight distribution can be initialized as desired and permitted to change or be fixed during training. For the majority of our experiments, input weights were initialized with a sparse uniform distribution ($\:min=0.0,\:max=0.4$) and all weights were permitted to change during training while overall sparsity of connectivity was maintained.

### Time

Trials all take place over time. We use a discrete time step $\:\delta\:t=1ms$ for all our SNN work. Input ($\:x$) is given to the SNN ms-by-ms, and output ($\:y$) is read ms-by-ms. Dynamics such as all neurons’ membrane potentials ($\:v$) and spikes ($\:z$) change over ms as well. The current ms time point is denoted as $\:t$, the next time point as $\:t+1$, and so on.

### ALIF model neuron

The spiking neuron model used for all units in the recurrent SNN is the ALIF model. ALIF units contain two hidden state variables – one for the membrane potential $\:v$ and one for the variable $\:a$ which governs the adaptive spike threshold $\:A$. Together they determine whether a unit $\:i$ spikes ($\:{z}_{i}^{t}=1$) or does not spike ($\:{z}_{i}^{t}=0$) at time point $\:t$.

Each unit’s membrane potential evolves over time according to the equation:

$$\:{v}_{i}^{t+1}=\alpha\:{(v}_{i}^{t}-{E}_{m})+\sum_{i\ne\:j}{W}_{ji}^{rec}{z}_{j}^{t}+\sum_{i}{W}_{i}^{input}{x}_{i}^{t+1}-{z}_{i}^{t}{(v}_{th}-{E}_{m})$$

where the resting membrane potential $\:{E}_{m}=$ -$\:70.6mV$,

$$\:\mathrm{t}\mathrm{h}\mathrm{e}\:\mathrm{b}\mathrm{a}\mathrm{s}\mathrm{e}\mathrm{l}\mathrm{i}\mathrm{n}\mathrm{e}\:\mathrm{t}\mathrm{h}\mathrm{r}\mathrm{e}\mathrm{s}\mathrm{h}\mathrm{o}\mathrm{l}\mathrm{d}\:{v}_{th}=\:-50.4mV,$$

the decay factor $\:\alpha\:={e}^{-\delta\:t/{\tau\:}_{m}}$, and $\:{\tau\:}_{m}=20ms$ is the membrane time constant.

All synaptic connections and inputs in our model are current-based. At time point $\:t+1$, SNN unit $\:i$ receives recurrent spiking input from its presynaptic units $\:j$ which just spiked ($\:{z}_{j}^{t}$, weighted according to$\:{\:W}_{ji}^{rec}$), and a subset also receive stimulus input ($\:{x}_{i}^{t+1}$, weighted according to $\:{W}_{i}^{input}$). This is summed across all presynaptic units and all stimulus input sources, so that the input current into unit $\:i$ at time $\:t+1$ is given as:

$$\:{I}_{i}^{t+1}=\sum_{i\ne\:j}{W}_{ji}^{rec}{z}_{j}^{t}+\sum_{i}{W}_{i}^{input}{x}_{i}^{t+1}$$

Finally, the term $\:{z}_{i}^{t}{(v}_{th}-{E}_{m})$ reduces a unit’s membrane potential by a constant value after neuron i spikes (Bellec et al. 2020). $\:{z}_{i}^{t}$is further fixed to be 0 for a refractory period of 4ms following unit i spiking.

By the above equations, all units’ membrane potentials evolve over time. When a unit’s membrane potential exceeds the spike threshold, that unit will emit a spike. For ALIF neurons, the spike threshold is adaptive, meaning it also evolves over time.

The adaptive threshold increases following a spike and decays exponentially to the baseline threshold $\:{v}_{th}$. This can be described by the equations:

$$\:{A}_{i}^{t}={v}_{th}+\beta\:{a}_{i}^{t}\:,\:\mathrm{w}\mathrm{h}\mathrm{e}\mathrm{r}\mathrm{e}\:\beta\:=0.16\:,$$

$$\:{z}_{i}^{t}=H({v}_{i}^{t}-{A}_{i}^{t}),$$

where $\:H$ is the Heaviside step function,

$$\:{a}_{i}^{t+1}=\rho\:{a}_{i}^{t}+{z}_{i}^{t},$$

$$\:\rho\:={e}^{-\delta\:t/{\tau\:}_{a}}\:,\:\mathrm{w}\mathrm{h}\mathrm{e}\mathrm{r}\mathrm{e}\:{\tau\:}_{a}=100ms,$$

which can be altered to fit timescales relevant to the task of interest (Bellec et al. 2020).

Essentially, if a unit’s membrane potential at time t exceeds its adaptive threshold at time t, the unit will emit a spike.

$$\:\mathrm{I}\mathrm{f}\:{v}_{i}^{t}\:>\:{A}_{i}^{t}\:,\:\mathrm{t}\mathrm{h}\mathrm{e}\mathrm{n}\:{z}_{i}^{t}=1.$$

At the very beginning when $\:t=0$, membrane potentials are initialized with a random normal distribution (µ = −65 mV, σ = 5 mV).

### Structure

SNNs are initialized with neocortical structural properties, some of which continue to be enforced during training. Initial excitatory weights follow a long-tailed, log-normal distribution (Song et al. 2005), where µ = −0.64 mV, σ = 0.51 mV, corresponding to a mean of 0.6005 mV and a standard deviation of 0.1071 mV (Bojanek & Zhu et al. 2020). Inhibitory weights follow the same distribution but with values − 10x stronger than excitatory.

All units are sparsely and recurrently connected; the precise probabilities of connection within and between e and i populations are taken from neocortical experiments: p_e→e_: 0.160, p_e→i_: 0.205, p_i→e_: 0.252, p_i→i_: 0.284 (Billeh et al. 2020). Weight values and connections are permitted to change during training. However, overall sparsity is maintained (details in “Sparsity and rewiring” Sect. 4.11).

### Dynamics

In addition to structural constraints, models can be constrained to exhibit spiking dynamics that match neocortical dynamics during training. For example, spiking in neocortex is sparse, asynchronous, and near-critical (Brunel 2000; Renart et al. 2010; Zerlaut et al. 2019), and each of these features can be maintained in the SNNs’ activity throughout training. For this study, we focus on enforcing sparse spiking during training, with a target of 20 Hz.

### Output

A subset of main SNN units sends weighted connections to the output. Thus, $\:{W}_{i}^{output}{z}_{i}^{t}$ is the output activation from SNN unit $\:i$ at time $\:t$. Output connectivity is initialized using the same statistics as the recurrent SNN’s connectivity, and sparsity is maintained during training in the same manner.

The initial recurrent weights supported biologically realistic, low spike rates in the recurrent layer, but the initial output weights prevented spiking activity from driving the output (Fig. 3A). This initialization encouraged the network to develop task-relevant weights through training. Only after training did the combined weights produce meaningful output activations (Fig. 3B).

### Training

SNNs are trained using BPTT with modifications for spiking. The goal of training is to reduce the difference between the SNNs’ output and the desired output, while still maintaining naturalistic dynamics and structure. This is achieved through iteratively modifying the weights of the recurrent SNN. Input and output weights are also permitted to change.

Since tasks are temporal, the task error (aka loss) at each timepoint is the mean squared error between the desired output (aka target) at time t and the SNNs’ actual output (aka prediction) at time t.

$$\:{E}_{task}^{t}=MSE({y}_{pred}^{t}\:,{y}_{target}^{t})$$

As the task and training take place over time, the total loss is summed over all time points $\:t$.

To maintain naturalistic sparse spiking during dual or rate-only training, the deviation (MSE) of the SNN’s actual spike rate from that target rate is added to the loss (Zhu et al. 2020).

$$\:{E}_{rate}=MSE({rate}_{SNN},\:{rate}_{target})$$

$$\:{E}_{total}={E}_{task}+{E}_{rate}$$

### Gradient descent with Adam optimizer

The goal of training is to reduce the total loss. To do so, we iteratively modify the weights in the network using a version of stochastic gradient descent.

The amount by which each weight should change in each iteration is determined by its gradient, which can be written as:

$$\:\frac{dE}{d{W}_{ij}}=\sum\:_{t}\frac{dE}{d{z}_{i}^{t}}*\frac{d{z}_{i}^{t}}{d{s}_{i}^{t}}*\frac{d{s}_{i}^{t}}{d{W}_{ij}}$$

The only new variable here is $\:{s}_{i}^{t}$, which is the hidden state of neuron $\:i$ at time $\:t$, and includes membrane potential and adaptation. $\:{s}_{i}^{t}=[{a}_{i}^{t},{v}_{i}^{t}]$.

Due to recurrent connectivity, the output, the hidden state of all units, and the spiking activity of all units at time $\:t$ is dependent on all other units’ states and activities at time $\:t-1$, and at time $\:t-2$, and so on to $\:t=0$. Those previous states and activities in turn depend on the weights. Therefore, terms are recursively expanded over past time steps (details in Bellec et al. 2020).

We use the Adam optimizer to determine precisely how weights should change based on the gradients. Typically in stochastic gradient descent, each gradient is multiplied by a static learning rate to yield the value by which each weight should change. The Adam optimizer instead adapts the learning rate for every variable over the course of training (details in Kingma and Ba 2014).

### Spike pseudo-derivative

The above is the standard form of BPTT for recurrent neural networks. However, because the units in our network spike, and spikes are not differentiable (recall that $\:{z}_{i}^{t}$ is determined by the Heaviside step function), we use a pseudo-derivative to replace the term $\:\frac{d{z}_{i}^{t}}{d{s}_{i}^{t}}$ (Huh and Sejnowski 2018; Bellec et al. 2020). Outside of the refractory period, the pseudo-derivative is defined as:

$$\:{\psi\:}_{i}^{t}=\frac{1}{{v}_{th}}{\gamma\:}_{pd}max(\mathrm{0,1}-|\frac{{v}_{i}^{t}-{A}_{i}^{t}}{{v}_{th}}\left|\right),\:\mathrm{w}\mathrm{h}\mathrm{e}\mathrm{r}\mathrm{e}\:{\gamma\:}_{pd}=0.3.$$

During the refractory period, $\:{\psi\:}_{i}^{t}=0$.

### Sparsity and rewiring

To maintain the overall level of connectivity in the SNN, all weights in all layers that are initialized as 0’s ($\:{W}_{ij}^{}=0$ indicates that units $\:i$ and $\:j$ are “disconnected”) will maintain their 0 values during training. All other weights can be updated via gradient descent. An exception occurs when an existing connection weight flips sign (e.g. an excitatory connection weight becomes negative) or becomes 0. In that case, that connection is set to 0 (e.g. is “pruned” away) and a new connection is randomly drawn from the pool of 0-valued connections. The new connection’s weight value is drawn from the initial weight distribution. This process is akin to synaptic rewiring in neocortex (Bellec et al. 2018).

### Task

Models were trained on a visual motion entropy change detection task. The model is presented with videos of 460 white drifting dots on a black background. Dots drift at a speed of 10 pixels per ms at two motion entropy levels. In the low entropy case, 100% of dots move together in one of four cardinal directions (0°, 90°, 180°, 270°). In the high entropy case, only 15% of dots move together in one of the cardinal directions; all other dots may move in any other direction, including non-cardinal ones. Dots are randomly placed on the screen at the start of each video trial and are initialized with random remaining durations to their lifetimes (total lifetime of 1000 ms). Each time a dot reaches its max lifetime, it is removed, and a new dot is randomly drawn at a new location.

Each video trial has a total duration of 4080 ms. Half of all trials have a change in motion entropy which occurs at a random time between 500 and 3500 ms within the trial. The task is for the model to report the motion entropy level at all timepoints of the trial.

### CNN front-end

**Fig. 13:**
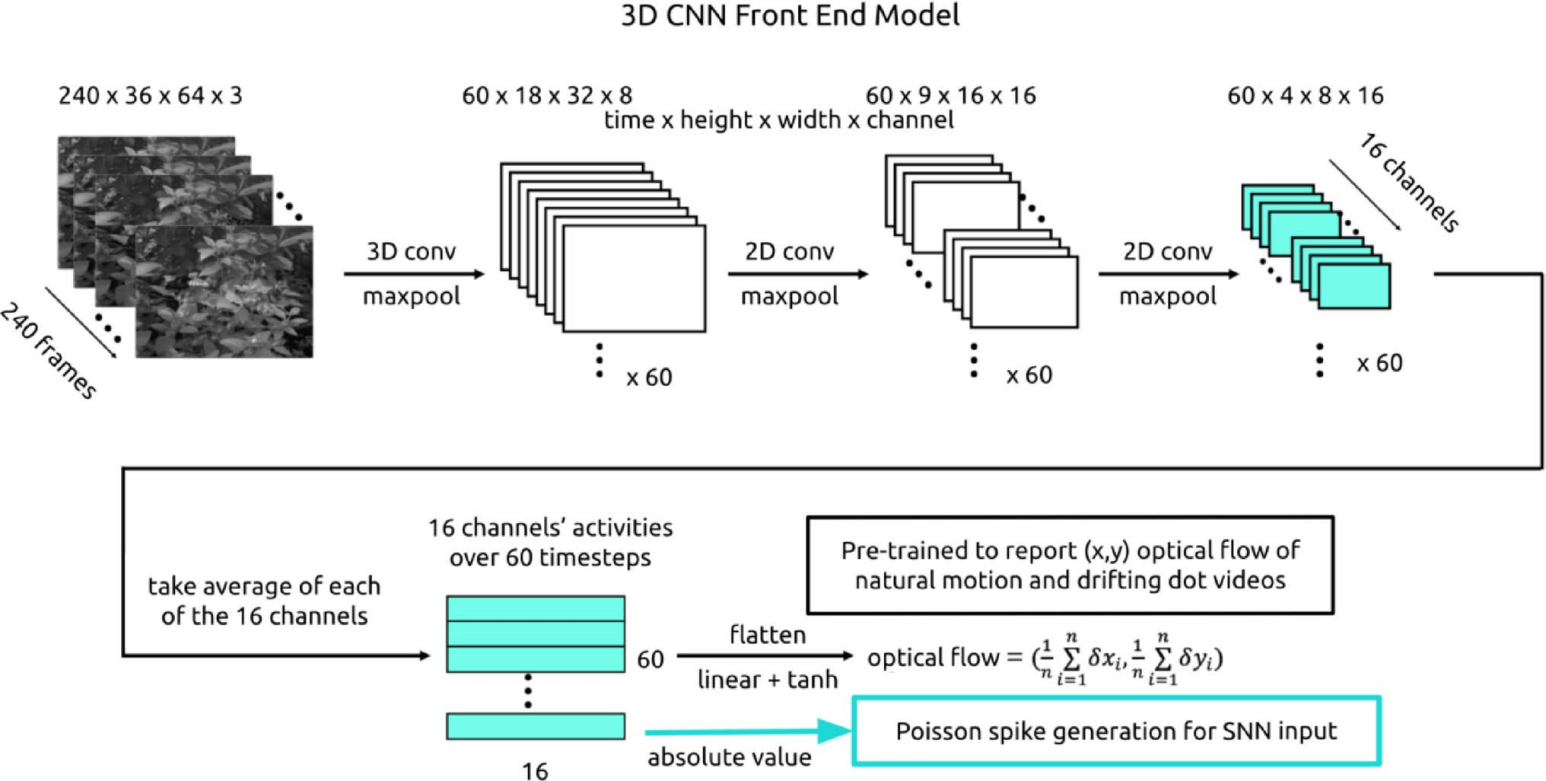
Model front-end architecture. To make videos interpretable to the main SNN, we built a 3D CNN front-end. The activations of the CNN’s last layer were interpreted as firing rates from which Poisson spikes were generated

To make the videos interpretable to the main SNN, we created and trained a 3D CNN model of retina and thalamus as a preprocessor that turns videos into spike sequences (Maheswaranathan et al. 2023). This CNN model was trained to report the velocity (x, y) of global motion in a combined dataset of drifting dot videos and black-and-white natural motion videos (Fig. 13).

The CNN model is composed of three successive blocks of Conv3D, MaxPooling3D, and dropout layers, after which the output is flattened into a prediction of x velocity and a prediction of y velocity. We take the average, absolute value activations of the 16 units in the last layer to indicate Poisson firing rates. Since the CNN compresses video input to 60 timesteps, we get a vector of 60 values from each of the 16 units. We expand this back to 4080 ms by repeating each of the 60 values 68 times. In other words, we assume the same underlying firing rate for each block of 68 ms. These rates are used to generate Poisson spikes that are given as input to a subset of recurrent SNN units.

### Input channel modulation

Input channels were positively modulated by either high motion or low motion entropy input. The difference between mean response rates to the two entropy levels (high entropy minus low entropy) are as follows for the 16 input channels: 0.0163, 0.0002, 0.0192, 0.0874, -0.0176, -0.0163, -0.0063, -0.0170, 0.0284, 0.0033, 0.0180, -0.0395, -0.0336, -0.0118, -0.0166, 0.0071 spikes/ms.

In the case of low motion entropy, input channels exhibited multimodal rate distributions (Fig. 7). In contrast, in response to high motion entropy, the input channels’ rate distributions were largely unimodal. When we consider medians instead of means, 10 input channels had greater median rates in response to high motion entropy, and 6 input channels had greater median rates in response to low motion entropy. The average distance between all medians was 0.023 ± 0.029 spikes/ms. When we consider modes, 5 input channels had greater modes in response to high motion entropy, and 6 input channels had greater modes in response to low motion entropy. Finally, 5 input channels had the same modes to both low and high motion entropy, further illustrating the similarity of input responses to the two motion entropy states. The average distance between all modes was 0.045 ± 0.048 spikes/ms.

We chose the mean as the measure of central tendency to define whether input channels were positively modulated by high or low motion entropy; the majority of low motion entropy rate distributions were trimodal, making the mean the best measure for comparison across all distribution shapes.

### Training sequence

A single trial involves an input sequence and a target output sequence. The input sequence is composed of Poisson spiking activity from 16 input units. The target sequence is 0’s and/or 1’s - the label attached to each motion entropy level is swapped in different experiments.

SNNs are trained on a large set of these trials. There are a total of 600 unique trials, which are repeated and shuffled to create the desired total training set size. Experiments were run for a duration of 30 trials per batch x 10,000 total batch updates, which yields 300,000 total trials.

To reduce overfitting, in which the SNN becomes overly good at particular trials but not others, we accumulate $\:{E}_{total}$ and $\:\frac{dE}{d{W}_{ij}}$ over a batch of 30 trials before updating weights. The process then repeats for the next batch of 30 trials. Due to trial shuffling and repetition, each batch contains a unique set of trials. In this way, weight changes will improve the average performance over many different trials.

### Software and hardware

All SNN training was completed using Python 3.8 or higher run on Nvidia GPUs with CUDA version 11.2 or higher.

### Statistical analysis

To compare all distributions of SNN measures, such as initial and trained firing rates to the two motion entropy levels, initial and trained weight distributions of input, recurrent, and output layers, etc., we performed two-sample KS testing and reported both the distance between means and the p-value.

### Weight changes according to modulation during dual training of the default network

**Table 4:** Weights changes according to modulation type

Connection type	Initial (mV)	Trained (mV)
e→e		
‘0’-modulated to ‘0’-modulated	0.021 ± 0.007	0.10 ± 0.05
‘1’-modulated to ‘1’-modulated	0.025 ± 0.009	0.128 ± 0.011
‘0’-modulated to ‘1’-modulated	0.024 ± 0.007	0.076 ± 0.015
‘1’-modulated to ‘0’-modulated	0.022 ± 0.007	0.056 ± 0.013
e→i		
‘0’-modulated to ‘0’-modulated	0.030 ± 0.014	0.19 ± 0.05
‘1’-modulated to ‘1’-modulated	0.027 ± 0.012	0.13 ± 0.02
‘0’-modulated to ‘1’-modulated	0.025 ± 0.010	0.10 ± 0.03
‘1’-modulated to ‘0’-modulated	0.024 ± 0.010	0.045 ± 0.004
i→e		
‘0’-modulated to ‘0’-modulated	-0.23 ± 0.08	-0.52 ± 0.10
‘1’-modulated to ‘1’-modulated	-0.24 ± 0.09	-0.61 ± 0.14
‘0’-modulated to ‘1’-modulated	-0.27 ± 0.10	-0.94 ± 0.16
‘1’-modulated to ‘0’-modulated	-0.27 ± 0.12	-1.0 ± 0.2
i→i		
‘0’-modulated to ‘0’-modulated	-0.23 ± 0.07	-0.41 ± 0.15
‘1’-modulated to ‘1’-modulated	-0.24 ± 0.06	-0.52 ± 0.14
‘0’-modulated to ‘1’-modulated	-0.28 ± 0.11	-0.9 ± 0.2
‘1’-modulated to ‘0’-modulated	-0.33 ± 0.14	-1.1 ± 0.3

### Weight changes during rate-only and task-only training of the single output network

A similar pattern of weight changes occurred during training on only rate as during dual training. Recurrent weights changed the most (*p* ≈ 0.0 for all naive and trained recurrent weight distributions). Inputs to excitatory units changed more than inputs to inhibitory units (in→e: *p* = 1.827 · 10^− 40^; in→i: *p* = 0.030). Output layer weights did not change (e→out: *p* = 1.0; i→out: *p* = 1.0).

After training on only task, recurrent weights also changed the most (*p* ≈ 0.0 for all naive and trained recurrent weight distributions), while input layer weights to inhibitory units (in→e: *p* = 3.137 · 10^− 17^; in→i: *p* = 0.077) and output layer weights (e→out: *p* = 0.202; i→out: *p* = 1.0) did not change.

## Data Availability

Modeling data is available at https://doi.org/10.5281/zenodo.17943749.
